# State tobacco control expenditures and tax paid cigarette sales

**DOI:** 10.1371/journal.pone.0194914

**Published:** 2018-04-13

**Authors:** John A. Tauras, Xin Xu, Jidong Huang, Brian King, S. Rene Lavinghouze, Karla S. Sneegas, Frank J. Chaloupka

**Affiliations:** 1 Department of Economics, University of Illinois at Chicago, Chicago, IL, United States of America; 2 Office on Smoking and Health, Centers for Disease Control and Prevention, Atlanta, GA, United States of America; 3 Department of Health Management and Policy, Georgia State University, Atlanta, GA, United States of America; 4 Division of Health Policy and Administration, University of Illinois at Chicago, Chicago, IL, United States of America; Legacy, Schroeder Institute for Tobacco Research and Policy Studies, UNITED STATES

## Abstract

This research is the first nationally representative study to examine the relationship between actual state-level tobacco control spending in each of the 5 CDC’s Best Practices for Comprehensive Tobacco Control Program categories and cigarette sales. We employed several alternative two-way fixed-effects regression techniques to estimate the determinants of cigarette sales in the United States for the years 2008–2012. State spending on tobacco control was found to have a negative and significant impact on cigarette sales in all models that were estimated. Spending in the areas of cessation interventions, health communication interventions, and state and community interventions were found to have a negative impact on cigarette sales in all models that were estimated, whereas spending in the areas of surveillance and evaluation, and administration and management were found to have negative effects on cigarette sales in only some models. Our models predict that states that spend up to seven times their current levels could still see significant reductions in cigarette sales. The findings from this research could help inform further investments in state tobacco control programs.

## Introduction

Tobacco use remains the single most preventable cause of disease and death in the United States[1]. In order to reduce tobacco use prevalence and prevent tobacco use initiation, education and state-wide and community-wide tobacco control initiatives have been implemented for decades. Over time, these initiatives have evolved into more comprehensive tobacco control programs, which are typically organized and funded at the state level[2]. Aimed at reducing tobacco related disease, disability, and death, these state programs usually involve functioning infrastructure, administrative and management support, as well as surveillance and evaluation[3–5].

Following the establishment of state comprehensive tobacco control programs in Minnesota in 1985 and California in 1989, Massachusetts, Arizona, and several other states also used ballot initiatives to pass tobacco excise tax increases and use a portion of the revenues generated from these taxes to fund tobacco control programs. Other states were successful in using the legislative process to establish tobacco control programs. A number of state tobacco control programs are funded by state settlements with cigarette manufacturers or by the funds states receive through the Master Settlement Agreement (MSA)[6]. Additionally, other funding for state tobacco control programs have come from private organizations and various federal sources.

It is estimated that the 50 states and the District of Columbia combined will collect $25.6 billion in fiscal year (FY) 2015 from tobacco tax revenues, MSA payments, and individual state settlements[7]. In the 2014 *Best Practice Guidelines for Comprehensive Tobacco Control Programs*, the Centers for Disease Control and Prevention (CDC) recommended that states spend a combined $3.3 billion, or $10.53 per capita, to maintain comprehensive tobacco control programs[5]. States have traditionally appropriated significantly less than what CDC recommends. In FY 2015, all 50 states and the District of Columbia combined appropriated $490.4 million for tobacco prevention and cessation programs[7]. The amount of funds allocated for tobacco control in FY 2015 was approximately 14.8% of the recommended level of funding, and is less than 2% of the revenue that states received from tobacco settlements and tax revenues. Moreover, the amount that states allocated for tobacco control in FY 2015 was considerably less than the amount spent just a few years ago. Across the country, state funding on tobacco control efforts has decreased by almost 32% since FY 2008[7]. In FY 2015, only Alaska and North Dakota funded tobacco control efforts at the recommended levels, and only five other states funded tobacco control efforts at least half of these levels[7].

In 1999, CDC issued its first *Best Practices for Comprehensive Tobacco Control Programs*, which provided guidance on nine key components of comprehensive state tobacco control programs and included recommendations for funding of these programs, largely based on evidence from California and Massachusetts[8]. In 2007, based on growing evidence from more states, the CDC revised its guidelines and updated its funding recommendations[4]. The revised guidelines detailed five essential components for comprehensive tobacco control programs using evidence of the effects of state tobacco control programs found in the scientific literature. The five components included: state and community interventions; health communication interventions; cessation interventions; surveillance and evaluation; and administration and management. The most recent revision to the *Best Practices for Comprehensive Tobacco Control Programs* was released in 2014[5]. The 2014 *Best Practices* updates the guidance provided in 2007 to reflect additional state evidence and experiences, new scientific literature, and changes in state populations, inflation, and the national tobacco control landscape since its previous release. The recommended funding level outlined in the report represents the annual level of investment for ensuring a fully funded and sustained comprehensive tobacco control program with resources sufficient to most effectively reduce tobacco use. The Administration and Management section also was renamed to the Infrastructure, Administration, and Management section to reflect the growing understanding of fully functioning and sustainable infrastructure for comprehensive tobacco control programs[7,9,10].

Evaluations of individual state programs provide compelling evidence that these programs are correlated with reduced tobacco use[11–13]. For example, after adopting large-scale comprehensive state tobacco control programs, Arizona, Florida, Massachusetts, Oregon, and California observed significant reductions in smoking[14–21]. However, many of these studies used relatively simple trend analyses that did not control for potential confounding factors. Another potential limitation of the state-specific evaluations is that the results might not be generalizable to other states or to the United States as a whole. In addition to these state level studies, a few national-level analyses have examined the impact of state tobacco control programs on cigarette smoking. These studies controlled for confounding factors and found comprehensive tobacco control programs to decrease cigarette sales[22,23], decrease youth smoking prevalence and smoking intensity[24], decrease college student smoking prevalence[25], and decrease adult smoking prevalence[26]. Our study builds upon these national studies, but unlike the previous studies based on state-specific tobacco control appropriations, our study utilizes actual tobacco control spending in each state in each of the five Best Practice categories for the years 2008–2012. It is the first national study to use these newly collected data on state tobacco control program spending to examine the relationship between actual state-level tobacco control spending in each of the CDC’s Best Practices categories and cigarette sales.

## Methods

### Data

#### Dependent variable (cigarette sales)

The dependent variable in all specifications was state aggregated per-capita cigarette sales. The cigarette sales data, which were obtained from the 2013 *Tax Burden on Tobacco*[27], represent annual tax-paid sale volumes between July 1 and June 30 for each fiscal year 2008–2012. The cigarette sales data were converted to per-capita sales using state level population estimates from the United States Census Bureau[28].

#### Independent variables

**Tobacco control expenditures:** Numerous independent variables were constructed and included in the models. Of particular importance were variables pertaining to tobacco control expenditures. Data on state comprehensive tobacco control expenditures were obtained from the Health Policy Center at the University of Illinois at Chicago; this new dataset collects actual tobacco control spending in each of the 5 Best Practice categories, by state, for the years 2008–2012. A detailed description of the data is available elsewhere[29]. Based on the state expenditure data, we created several variables relative to CDC recommendations for each state using 2007 CDC *Best Practice* recommended funding levels. We used the 2007 recommended funding levels in this analysis because these were the funding guidelines for states during the time period examined. The 2007 CDC recommendations were adjusted for inflation and population each year using the Bureau of Labor Statistics Current Price Index and population data from the US Census Bureau, respectively. We first created a set of indicators representing the percent of 2007 CDC funding recommendations that each state spent on tobacco control, using <25% as the referent: 25% <50%, 50% — <75%, and ≥75%. We also created a continuous measure representing the percent of 2007 funding recommendations spent on tobacco control. To account for a likely non-linear effect of tobacco control spending on cigarette demand, we also created a quadratic term for the percent of recommended funding level spent on tobacco control variable.

We also created inflation-adjusted real per capita measures of spending on tobacco control for each state in each fiscal year. We created a variable reflecting the real total per capita spending on tobacco control overall and the real total per capita spending in each of the five 2007 *Best Practice* categories, including: health communication interventions; cessation interventions; state and community interventions; surveillance and evaluation; and administration and management. Again, we also created quadratic variables for real per capita total spending and each of the aforementioned real per capita spending categories.

#### Annual cigarette price*s*

The annual *Tax Burden on Tobacco*[27] which provides annual cigarette price as of November 1 for each year, was used to create a weighted average state price for each fiscal year. The weighed state price accounted for the timing of the April 1, 2009 Federal tax increase, as well as state cigarette tax changes that occurred throughout this period. To account for inflation, all cigarette prices were deflated using the national Consumer Price Index (CPI) published by the Bureau of Labor Statistics[30].

#### Smoke-free law coverage

We also created three variables that reflect the percent of the state population covered by a smoke-free law prohibiting smoking in private worksites, restaurants, and bars, respectively, taking into account both state and local level laws in each fiscal year. Following Huang et al. (2015), we then created an index variable reflecting the average of these three variables to be used in our models[31].

**Unemployment and income:** From the Bureau of Labor Statistics, we obtained monthly unemployment rate data for each state and the District of Columbia (DC)[32]. We converted the monthly data into average fiscal year unemployment numbers. From the United States Department of Commerce–Bureau of Economic Analysis, we obtained quarterly state specific personal income data for each state and DC. We converted the quarterly data into fiscal year data and deflated by the national Consumer Price Index published by the Bureau of Labor Statistics (1982–1984 = 100) to adjust for inflation.

**Population demographic factors:** From the United States Census Bureau, we obtained state level population data as of July 1 of each year[29]. We defined variables that represent: the total state population; the percent of the population aged less than 5 years, 5–17 years, 18–24 years, 25–44 years, 45–64 years, and 65 years or older; the percent of the population that is non-Hispanic White, Hispanic, non-Hispanic Black, non-Hispanic American Indian or Alaskan Native, and non-Hispanic other race/ethnicity. Finally, from the United States Census Bureau, we obtained the percent of each state’s population that has less than a high school degree, a high school degree or some college, and a Bachelor’s degree or more[33].

Finally, Table 1 provides the descriptive statistics for the variables used in the analyses.

**Table 1:** Descriptive statistics: United States for years 2008–2012.

Variable	Mean	SD^†^	Min^†^	Max^†^
Per Capita Cigarette Sales	54.42	22.49	17.32	139.98
Real Cigarette Price	5.67	1.22	3.52	9.99
State Tobacco Control expenditure measures				
Real Per Capita Total Expenditure	3.59	3.35	0.07	16.87
Real Per Capita Expenditure–Health Communication	0.64	0.65	0.00	3.05
Real Per Capita Expenditure–Cessation	0.81	0.87	0.02	4.09
Real Per Capita Expenditure–State and Community	1.49	1.57	0.00	8.08
Real Per Capita Expenditure–Surveillance and Evaluation	0.28	0.37	0.00	2.05
Real Per Capita Expenditure–Administration	0.38	0.39	0.00	1.93
Spending > = 25% but < 50% of CDC Recommendation	0.28	0.45	0.00	1.00
Spending > = 50% but < 75% of CDC Recommendation	0.06	0.24	0.00	1.00
Spending > = 75% of CDC Recommendation	0.04	0.18	0.00	1.00
Percent of CDC Recommendation	23.96	19.65	0.50	94.9
State level control variables				
% of Pop SFA	0.61	0.38	0.00	1.00
Unemployment Rate	7.60	2.17	3.03	13.80
Real Income	41,015.6	7,430.6	30,249	74,773
Male	49.21	0.75	47.08	51.98
Race/ethnicity				
Non-Hispanic White	79.91	13.32	25.48	95.97
Hispanic	10.59	9.79	1.21	46.98
Non-Hispanic Black	11.47	11.02	0.43	53.68
Non-Hispanic AI/AN	1.89	2.93	0.21	14.97
Non-Hispanic Other Race	6.72	9.78	1.79	72.52
Education				
Less than High School	13.18	3.38	8.20	20.40
High School Degree/Some College.	59.28	5.20	37.6	68.1
College Degree	27.54	5.52	17.1	48.5
Age				
Age 5–17	17.20	1.45	11.18	22.11
Age 18–24	9.98	0.83	8.63	14.42
Age 25–44	26.11	1.72	23.15	35.48
Age 45–64	26.78	1.80	19.68	31.21
Age 65+	13.41	1.69	7.48	18.24

Note: SD = standard deviation; Min = minimum value of variable; Max = maximum value of variable

### Statistical analyses

We employed several alternative two-way fixed-effects regression techniques in the analyses. These fixed effects control for year-specific and state specific determinants of cigarette sales. The fixed effects approach included dichotomous indicators for years and states.

To assess the assumption of linearity we estimated an ordinary least squares regression and examined the residuals of the regression. First, we graphed the standardized residuals against the values of the tobacco control spending variable. Next we graphed an augmented component-plus-residual plot (i.e. augmented partial residual plot) as described by Mallows (1986) using locally weighted scatter plot smoothing[34]. Both plots show clear deviations from linearity. We used several approaches to deal with this nonlinearity. First, we employed ordinary least squares and a quadratic specification that allows for the possibility of diminishing returns to tobacco control spending. A limitation of the quadratic functional form is that the relationship between tobacco control spending and cigarette consumption must reach a maximum effect after which a positive relationship between tobacco control spending and cigarette consumption will occur. Second, we estimated a fixed-effects panel threshold model (FEPTM) developed by Hansen (1999)[35]. The FEPTM model examines the effect of tobacco control spending on sales and searches for a structural break in the relationship between the variables (i.e. a threshold level), at which point the relationship becomes less pronounced or changes sign. Unlike the quadratic specification, the FEPTM does not require the relationship between spending and consumption to reach an extreme value after which the direction of the effect changes. Specifically, we fit a single-threshold FEPTM model using 300 bootstrap replications and a default trimming proportion of 0.01 for all the equations except the health communication interventions and the surveillance and evaluation intervention equations where we used trimming proportions of 0.06 and 0.03, respectively, in order to be able to computationally estimate the thresholds. Overall, we fit three sets of models. For the first set of models (Table 2), Model 1 was estimated using ordinary least squares and contains the following covariates: real cigarette price, categorical indicators of state spending relative to CDC recommendations, smoke-free air index, unemployment rate, and real personal income, as well as variables reflecting gender, race/ethnicity, education, age distribution, and year and state fixed effects. Model 2 was identical to Model 1, except Model 2 replaced the indicator variables of spending relative to CDC recommended funding with the continuous and quadratic percent of CDC recommended funding variables. Model 3 was identical to Model 2, except Model 3 was estimated using the FEPTM model and excluded the quadratic term for the percent of CDC recommended funding.

**Table 2:** Impacts of state tobacco control expenditures on Per Capita cigarette sales: United States for years 2008–2012.

Variable	Model 1	Model 2	Model 3
Real Cigarette Price	-4.34 (-4.00)	-4.19 (-3.84)	-4.03 (-4.11)
State Tobacco Control expenditure measures			
Spending ≥ 25% but < 50% of CDC Recommendation	-2.86 (-2.26)		
Spending ≥50% but < 75% of CDC Recommendation	-4.55 (-2.20)		
Spending ≥75% of CDC Recommendation	2.48 (0.82)		
Percent of CDC Recommendation		-0.24 (-2.54)	
Percent of CDC Recommendation Squared		0.0028 (2.39)	
Estimated Threshold			76.7%
Percent of CDC Recommendation (% of CDC Recommendation < = 76.7%)			-0.065 (-1.71)
Percent of CDC Recommendation (% of CDC Recommendation >76.7%)			0.29 (4.79)
State level control variables			
% of Population Covered by Smoke-Free Air Laws	2.71 (1.60)	2.86 (1.68)	1.72 (1.11)
Unemployment Rate	0.06 (0.12)	0.14 (0.26)	-0.13 (-0.28)
Real Income	0.00 (1.22)	0.00 (1.72)	0.0008 (2.39)
Sex			
Male	-6.38 (-1.15)	-4.47 (-0.78)	-7.29 (-1.45)
Race/ethnicity			
Hispanic	2.15 (1.21)	2.19 (1.23)	3.23 (2.00)
Non-Hispanic Black	1.69 (0.86)	0.66 (0.33)	1.38 (0.78)
Non-Hispanic American Indian/Alaska Native	6.29 (1.16)	7.20 (1.30)	7.51 (1.53)
Non-Hispanic Other Race	3.48 (1.51)	3.58 (1.55)	3.72 (1.83)
Education			
High School Degree/Some College	-1.05 (-0.49)	-0.69 (-0.32)	-1.90 (-0.95)
College Degree	-2.06 (-0.96)	-1.85 (-0.85)	-1.49 (-0.77)
Age (years)			
5–17	-2.47 (-0.61)	-0.12 (-0.03)	-1.12 (-0.31)
18–24	2.30 (0.49)	2.83 (0.60)	4.37 (1.03)
25–44	5.42 (1.02)	5.38 (1.00)	4.91 (1.01)
45–64	3.34 (0.71)	3.33 (0.70)	2.59 (0.61)
65+	0.95 (0.23)	2.20 (0.53)	-0.10 (-0.03)
Year			
2009	-2.74 (-1.24)	-2.96 (-1.32)	-1.32 (-0.66)
2010	-6.08 (-2.11)	-6.57 (-2.24)	-4.95 (-1.90)
2011	-10.34 (-3.11)	-11.02 (-3.28)	-9.86 (-3.29)
2012	-13.44 (-3.10)	-14.63 (-3.34)	-13.27 (-3.38)

*Note*: All equations include an intercept and dichotomous indicators for each state in the sample minus 1. Regression coefficients are presented in the table and asymptotic *t* ratios are in parentheses. The critical values for the *t* ratios are 2.58 (2.33), 1.96 (1.64), and 1.64 (1.28) at the 1%, 5%, and 10% significance levels, respectively, based on a 2-tailed (1-tailed) test.

For the second set of models (Table 3), Model 1 was estimated using ordinary least squares and contained the following covariates: real cigarette price, continuous overall real per capita total spending on tobacco control, quadratic overall real per capita total spending on tobacco control, smoke-free air index, unemployment rate, and real personal income, as well as variables reflecting gender, race/ethnicity, education, age distribution, and year and state fixed effects. Models 2–6 were identical to Model 1, except Models 2–6 replaced the continuous and quadratic overall real per capita total spending on tobacco control variables with the real per capita spending for each of the 5 Best Practice categories separately using both a continuous and quadratic term in the models.

**Table 3:** Impacts of state tobacco control expenditures by Best Practice category on per capita cigarette sales: United States for years 2008–2012 quadratic model.

Variable	Model 1	Model 2	Model 3	Model 4	Model 5	Model 6
Real Cigarette Price	-4.29 (-4.00)	-4.10 (-3.83)	-4.25 (-3.82)	-3.79 (-3.47)	-4.12 (-4.00)	-4.14 (-3.82)
State Tobacco Control expenditure measures						
Real Per Capita Total Expenditure	-1.88 (-3.29)					
Real Per Capita Total Expenditure Squared	0.140 (3.53)					
Real Per Capita Expenditure–Health Communication		-5.16 (-2.53)				
Real Per Capita Expenditure–Health Communication Squared		2.80 (3.57)				
Real Per Capita Expenditure–Cessation			-3.57 (-1.96)			
Real Per Capita Expenditure–Cessation Squared			0.753 (1.74)			
Real Per Capita Expenditure–State and Community				-1.11 (-1.00)		
Real Per Capita Expenditure–State and Community Squared				0.053 (0.32)		
Real Per Capita Expenditure–Surveillance and Evaluation					-13.28 (-3.88)	
Real Per Capita Expenditure–Surveillance and Evaluation Squared					9.24 (5.00)	
Real Per Capita Expenditure–Administration and management						-9.44 (-2.14)
Real Per Capita Expenditure–Administration and management Squared						7.18 (2.75)
State level control variables						
% of Population Covered by Smoke-Free Air Laws	2.93 (1.75)	2.07 (1.24)	2.70 (1.57)	2.92 (1.69)	2.39 (1.47)	2.30 (1.35)
Unemployment Rate	0.17 (0.31)	0.29 (0.55)	0.04 (0.07)	0.08 (0.15)	0.08 (0.15)	0.31 (0.58)
Real Personal Income	0.00 (1.89)	0.00 (2.46)	0.00 (1.83)	0.00 (1.95)	0.00 (1.83)	0.00 (1.83)
Sex						
Male	-3.81 (-0.69)	-8.57 (-1.59)	-6.55 (-1.18)	-7.98 (-1.39)	-7.04 (-1.34)	-9.77 (-1.74)
Race/ethnicity						
Hispanic	2.39 (1.36)	2.40 (1.38)	2.38 (1.34)	2.69 (1.47)	2.74 (1.63)	2.05 (1.17)
Non-Hispanic Black	0.28 (0.14)	-1.08 (-0.54)	1.65 (0.82)	1.50 (0.74)	2.39 (1.28)	1.49 (0.77)
Non-Hispanic American IndianAlaska Natives	8.44 (1.54)	6.72 (1.26)	5.67 (1.03)	3.49 (0.60)	7.24 (1.39)	2.55 (0.46)
Non-Hispanic Other Race	2.77 (1.21)	3.07 (1.37)	4.37 (1.91)	4.39 (1.86)	4.38 (2.06)	4.31 (1.92)
Education						
High School Degree/Some College	-0.67 (-0.31)	-1.59 (-0.74)	-0.77 (-0.34)	-1.10 (-0.50)	-1.32 (-0.63)	-2.37 (-1.07)
College Degree	-1.58 (-0.74)	-2.51 (-1.20)	-2.55 (-1.18)	-2.95 (-1.35)	-2.54 (-1.24)	-3.77 (-1.74)
Age (years)						
5–17	-0.04 (-0.01)	0.10 (0.03)	-0.31 (-0.07)	-0.57 (-0.14)	-1.89 (-0.50)	-0.05 (-0.01)
18–24	2.32 (0.50)	6.26 (1.34)	4.52 (0.96)	4.69 (0.97)	3.24 (0.72)	7.47 (1.57)
25–44	5.00 (0.94)	9.83 (1.81)	6.53 (1.20)	6.64 (1.21)	6.01 (1.18)	10.34 (1.88)
45–64	3.63 (0.77)	5.87 (1.25)	3.25 (0.67)	2.41 (0.51)	1.83 (0.41)	4.51 (0.95)
65+	2.59 (0.64)	4.27 (1.05)	1.78 (0.43)	1.15 (0.28)	0.52 (0.13)	2.97 (0.73)
Year						
2009	-3.23 (-1.47)	-2.98 (-1.39)	-1.98 (-0.88)	-2.12 (-0.94)	-2.04 (-0.98)	-2.07 (-0.95)
2010	-7.01 (-2.44)	-6.18 (-2.21)	-5.23 (-1.77)	-5.50 (-1.86)	-5.48 (-2.00)	-4.48 (-1.55)
2011	-11.62 (-3.52)	-11.04 (-3.42)	-10.02 (-2.96)	-10.02 (-2.97)	-9.65 (-3.06)	-8.70 (-2.61)
2012	-15.37 (-3.57)	-14.83 (-3.50)	-13.62 (-3.09)	-13.49 (-3.06)	-13.12 (-3.18)	-11.93 (-2.73)

*Note*. All equations include an intercept and dichotomous indicators for each state in the sample minus 1. Regression coefficients are presented in the table and asymptotic *t* ratios are in parentheses. The critical values for the *t* ratios are 2.58 (2.33), 1.96 (1.64), and 1.64 (1.28) at the 1%, 5%, and 10% significance levels, respectively, based on a 2-tailed (1-tailed) test.

The third set of models (Table 4) were identical to the second set of models except the third set of models were estimated using FEPTM regressions which did not include a quadratic term for tobacco control spending in any of the models. For all models, p<0.05 was the threshold used to establish statistical significance.

**Table 4:** Impacts of state tobacco control expenditures by Best Practice category on per capita cigarette sales: United States for years 2008–2012 fixed effects panel threshold model.

	Model 1	Model 2	Model 3	Model 4	Model 5	Model 6
Real Cigarette Price	-4.34 (-4.32)	-3.80 (-3.52)	-4.25 (-3.85)	-3.92 (-3.64)	-3.86 (-3.56)	-4.08 (-3.79)
State Tobacco Control expenditure measures						
Estimated Threshold	$12.52	$1.18	$3.02	$5.85	$0.06	$0.21
Real Per Capita Total Expenditure (Real Per Capita Total Expenditure< = $12.51)	-0.68 (-2.48)					
Real Per Capita Total Expenditure (Real Per Capita Total Expenditure>$12.51)	0.56 (2.01)					
Real Per Capita Expenditure–Health Communication (Real Per Capita Expenditure–Health Communication< = $1.18)		-1.70 (-1.13)				
Real Per Capita Expenditure–Health Communication (Real Per Capita Expenditure–Health Communication>$1.18)		0.93 (1.08)				
Real Per Capita Expenditure–Cessation (Real Per Capita Expenditure–Cessation< = $3.02)			-2.17 (-2.14)			
Real Per Capita Expenditure–Cessation (Real Per Capita Expenditure–Cessation>$3.02)			-0.48 (-0.59)			
Real Per Capita Expenditure–State and Community (Real Per Capita Expenditure–State and Community< = $5.85)				-1.48 (-2.61)		
Real Per Capita Expenditure–State and Community (Real Per Capita Expenditure–State and Community>$5.85)				-0.62 (-1.27)		
Real Per Capita Expenditure–Surveillance and Evaluation (Real Per Capita Expenditure–Surveillance and Evaluation< = $0.06)					46.3 (2.06)	
Real Per Capita Expenditure–Surveillance and Evaluation (Real Per Capita Expenditure–Surveillance and Evaluation>$0.06)					2.23 (1.27)	
Real Per Capita Expenditure–Administration and management (Real Per Capita Expenditure–Administration and management < = $0.21)						19.3 (2.95)
Real Per Capita Expenditure–Administration and management (Real Per Capita Expenditure–Administration and management >$0.21)						3.02 (1.46)
State level control variables						
% of Population Covered by Smoke-Free Air Laws	2.16 (1.37)	2.54 (1.51)	2.52 (1.47)	2.81 (1.66)	2.82 (1.65)	2.42 (1.42)
Unemployment Rate	-0.071 (-0.14)	0.28 (0.52)	0.12 (0.22)	0.13 (0.25)	0.094 (0.18)	0.14 (0.27)
Real Personal Income	0.0011 (3.09)	0.00062 (1.64)	0.00065 (1.72)	0.00077 (2.05)	0.00081 (2.12)	0.00087 (2.29)
Sex						
Male	-7.03 (-1.37)	-4.70 (-0.83)	-5.52 (-0.99)	-7.87 (-1.42)	-5.80 (-1.05)	-7.28 (-1.33)
Race/ethinicity						
Hispanic	3.88 (2.32)	1.59 (0.91)	2.17 (1.23)	2.32 (1.29)	2.67 (1.50)	1.48 (0.84)
Non-Hispanic Black	2.05 (1.12)	0.60 (0.31)	1.69 (0.84)	1.35 (0.69)	1.05 (0.54)	1.07 (0.55)
Non-Hispanic American IndianAlaska Natives	6.75 (1.34)	7.87 (1.42)	10.2 (1.72)	6.06 (1.07)	4.32 (0.79)	3.05 (0.56)
Non-Hispanic Other Race	3.69 (1.77)	5.06 (2.29)	3.83 (1.67)	3.99 (1.77)	4.85 (2.16)	4.28 (1.92)
Education						
High School Degree/Some College	-0.37 (-0.18)	-1.42 (-0.65)	-0.70 (-0.32)	-1.22 (-0.56)	-1.55 (-0.70)	-1.58 (-0.73)
College Degree	-1.84 (-0.93)	-2.95 (-1.39)	-2.20 (-1.02)	-2.81 (-1.32)	-3.06 (-1.42)	-3.29 (-1.54)
Age (years)						
5–17	-0.97 (-0.26)	-0.46 (-0.11)	-0.35 (-0.09)	-1.42 (-0.35)	-0.29 (-0.07)	0.066 (0.02)
18–24	3.08 (0.71)	4.51 (0.93)	4.44 (0.95)	3.16 (0.67)	4.42 (0.94)	5.89 (1.27)
25–44	5.19 (1.04)	7.72 (1.39)	6.02 (1.11)	5.63 (1.05)	7.31 (1.36)	8.86 (1.65)
45–64	2.31 (0.53)	3.84 (0.80)	3.15 (0.66)	1.92 (0.41)	3.61 (0.76)	4.49 (0.95)
65+	1.61 (0.42)	3.28 (0.79)	0.81 (0.20)	1.02 (0.25)	2.87 (0.70)	3.16 (0.78)
Year						
2009	-2.00 (-0.97)	-3.43 (-1.57)	-2.36 (-1.05)	-2.28 (-1.04)	-2.45 (-1.11)	-1.74 (-0.80)
2010	-6.02 (-2.26)	-6.53 (-2.28)	-5.31 (-1.81)	-5.87 (-2.05)	-6.08 (-2.09)	-4.65 (-1.62)
2011	-11.6 (-3.76)	-11.0 (-3.34)	-9.78 (-2.91)	-10.4 (-3.14)	-11.2 (-3.33)	-9.62 (-2.92)
2012	-16.4 (-4.05)	-14.6 (-3.40)	-12.8 (-2.92)	-13.9 (-3.22)	-15.2 (-3.44)	-13.2 (-3.07)

*Note*. All equations include an intercept and dichotomous indicators for each state in the sample minus 1. Regression coefficients are presented in the table and asymptotic *t* ratios are in parentheses. The critical values for the *t* ratios are 2.58 (2.33), 1.96 (1.64), and 1.64 (1.28) at the 1%, 5%, and 10% significance levels, respectively, based on a 2-tailed (1-tailed) test.

## Results

### Overall findings

States that spent at least 25%, but less than 75%, of the CDC recommended levels had significantly lower per capita cigarette sales than states that spent <25% of CDC recommended levels (p<0.05). No significant differences were found for those states that spent ≥75% of the CDC recommendation on tobacco control and those states that spent <25% of the CDC recommendation. The ≥75% finding should be viewed with caution because very few states over this time spent ≥75% of the CDC recommendation on tobacco control. Between FY2008 and FY2012, an average of 32.0, 14.1, 3.0, and 1.8 states per year spent <25%, > = 25% but < 50%, > = 50% but <75%, and ≥75% of the CDC recommendation on tobacco control, respectively (Table 1). Moreover, between FY2008 and FY2012, the average inflation adjusted total per capita expenditure on tobacco control for states that spent <25%, > = 25% but < 50%, > = 50% but <75%, and ≥75% of the CDC recommendation was $1.65, $5.26, $10.17, and $13.83, respectively. Furthermore, between FY2008 and FY2012, the average per capita cigarette sales for states that spent <25%, > = 25% but < 50%, > = 50% but <75%, and ≥75% of the CDC recommendation was 57.4, 45.4, 63.2, and 58.2, respectively.

Using the quadratic specification in Model 2, we find a non-linear relationship between the percent of CDC recommended spending on tobacco control and per capita cigarette sales (p<0.05). The coefficient estimates indicate that per capita cigarette sales decreased as the percentage of CDC recommended spending increased, until the percentage of CDC recommended spending increased to approximately 42.9% (Table 2). On the other hand, in Model 3 where we used the FEPTM regression, we find that per capita cigarette sales decreased as the percentage of CDC recommended spending increased, until the percentage of CDC recommended spending increased to approximately 76.7%.

Similarly, using the quadratic specification we find a nonlinear relationship between total per capita expenditures and per capita cigarette sales (p<0.01). The coefficient estimates indicate that per capita cigarette sales decreased as the total per capita spending on tobacco control increased until approximately $6.714 was spent per capita. This is significantly higher than the combined estimated per capita expenditures of $1.52 per capita states made in FY2014 (Table 3). Using the FEPTM (Table 4) we find increases in total per capita tobacco control spending decrease per capita cigarette sales until $12.52 per capita is spent on tobacco control. The estimate from the FEPTM regression is nearly double the estimate of the quadratic specification and implies that states could spend more than seven times their current levels before reductions in cigarette sales stop declining.

### Component specific findings–quadratic specifications (Table 3)

Examining the actual real per capita tobacco control spending in each state in each of the five CDC *Best Practice* categories separately, we found real per capita expenditures on health communication interventions, cessation interventions, surveillance and evaluation, and administration and management to have a negative and significant impact on per capita cigarette sales, with the quadratic terms for per capita spending in each of these categories being positive (p<0.05, Table 3). The calculated per capita spending peaks were: health communication interventions ($0.92), cessation interventions ($2.96), surveillance and evaluation ($0.72), and administration and management ($0.66). Real per capita expenditures on state and community interventions were negative, but not statistically significant.

### Component specific findings–FEPTM regressions (Table 4)

Examining the actual real per capita tobacco control spending in each state in each of the five CDC *Best Practice* categories separately, we found real per capita expenditures on health communication interventions, cessation interventions, and state and community interventions to have negative impacts on per capita cigarette sales. Unlike the quadratic specification results, we found no negative effects of spending on surveillance and evaluation or administration and management on per capita cigarette sales. The threshold estimates imply that per capita sales of cigarettes will continue to decline until $1.18 per capita, $3.02 per capita, and $5.85 is spent on health communication interventions, cessation interventions, and state and community interventions, respectively.

## Discussion

Our results indicate that state spending on tobacco control initiatives is significantly associated with decreased cigarette sales. Spending in the areas of cessation interventions and health communication interventions were found to significantly decrease cigarette sales in all models that were estimated. Spending in the area of state and community interventions was found to significantly decrease cigarette sales in the FEPTM regression, but was found not to have a significant effect on sales in the quadratic specification. Mixed results were found for the effects of spending on surveillance and evaluation and on administration and management. The quadratic specification found spending in these areas to have a significant negative effect on sales, whereas the FEPTM model found no negative results for spending in these areas.

Our models predict that states that spend up to seven times their current levels could still see significant reductions in cigarette sales.

The validity of these findings is supported by the fact that the estimated per capita spending limits were consistent with the annual per capita funding levels for state programs in the 2014 *Best Practices*, although our data and analytical approach differ substantially.^5^ The overall minimum and recommended funding levels for comprehensive state programs are between $7.41 and $10.53 per capita in *Best Practices*[5], while our estimated spending per capita threshold is $6.71 using the quadratic model and $12.52 using the FEPTM. Similarly, the minimum funding levels by *Best Practice* category are $1.18, $2.53, $0.65, and $0.32 for mass-reach health communication interventions, cessation interventions, surveillance and evaluation, and infrastructure, administration and management respectively, while these figures are estimated around $0.92, $2.96, $0.72, and $0.66 using the quadratic model in our analysis. The FEPTM regressions indicate that the thresholds for health communication interventions, cessation interventions, and state and community interventions are $1.18, $3.02, and $5.85, respectively.

However, it is important to note that our estimated spending peaks should not be interpreted as the “optimal” levels of funding for state tobacco control programs. The estimated spending peaks presented in this analysis critically depend on the historical use of the funds and tobacco control and prevention interventions implemented by state tobacco control programs within each *Best Practice* category during the time period. For example, we find mixed results with respect to state and community interventions, surveillance and evaluation, and administration and management. These findings do not imply that decision makers should not allocate funds to such interventions. Rather, a number of factors, including but not limited to, a strong commitment, clear leadership, dedicated resources, and most importantly, applying evidence based population-level interventions, can affect the impact of the spending and might be investigated to facilitate program effectiveness. This could also include a commitment to fully functioning program infrastructure, as outlined in the Component Model of Infrastructure in the 2014 *Best Practices*[5]. Program infrastructure is the foundation that supports program capacity, implementation, and sustainability[9,10].

The findings in this study are subject to several limitations. First, the outcome measures used in the analysis are aggregate, state per-capita cigarette sales, and thus may not adequately reflect impacts of state tobacco control expenditures on individuals’ cigarette smoking behaviors. Second, we were unable to examine the impact of tobacco control expenditures separately by socioeconomic and demographic characteristics, and the potential differential impact for important subpopulations, such as youth, racial/ethnic/sexual minorities, those with low incomes, and those with mental health issues. Third, the analytical time frame for this analysis is relatively short, which limits the amount of variations in annual cigarette sales and state tobacco control expenditures and thus our ability to detect effects. Finally, state tobacco control expenditures is an aggregate measure of all tobacco control activities. Therefore, our estimated impact of tobacco control expenditures might be confounded by other state contextual factors related to conventional cigarette sales. We have partly addressed the issue by including cigarette per pack prices and the percentages of state population covered by comprehensive smoke-free air laws.

In conclusion, the findings from this study provide supporting evidence that sustained funding for state tobacco control programs is associated with reduced cigarette consumption. These findings, along with previous work, could help to inform state decision making about investments in state tobacco control programs and implementation of evidence based strategies. Additional research demonstrates the cost-effectiveness of investments in state tobacco control programs, with one recent study by Lightwood and Glantz (2013) concluding that the $2.4 billion invested in California's tobacco control program between FY1989 and FY2008 led to a cumulative reduction of $134 billion in health care spending in the state [36]. Despite this evidence, most states fund their tobacco control programs at levels well below recommended levels. The 2014 *Best Practices* recommended that states allocate a total of $3.3 billion to their comprehensive tobacco control programs, but total state investments were $490.4 million in FY2015 [7]. Based on the findings of this study, fully funded and sustained comprehensive tobacco control programs with sufficient resources could lead to significant reductions in death, disease, and economic consequences caused by tobacco use.
